# *Mycoplasma pneumoniae* Outbreak in a Long-Term Care Facility — Nebraska, 2014

**Published:** 2015-03-27

**Authors:** Deborah L. Hastings, Kari J. Harrington, Preeta K. Kutty, Rebecca J. Rayman, Dana Spindola, Maureen H. Diaz, Kathleen A. Thurman, Jonas M. Winchell, Thomas J. Safranek

**Affiliations:** 1 Epidemic Intelligence Service, CDC; 2 Nebraska Department of Health and Human Services; 3 East Central District Health Department, Nebraska; 4 National Center for Immunization and Respiratory Diseases, CDC

On June 20, 2014, a Nebraska long-term care facility notified the East Central District Health Department (ECDHD) and Nebraska Department of Health and Human Services (NDHHS) of an outbreak of respiratory illness characterized by cough and fever in 22 residents and resulting in four deaths during the preceding 2 weeks. To determine the etiologic agent, identify additional cases, and implement control measures, Nebraska and CDC investigators evaluated the facility’s infection prevention measures and collected nasopharyngeal (NP) and oropharyngeal (OP) swabs or autopsy specimens from patients for real-time polymerase chain reaction (PCR) testing at CDC. The facility was closed to new admissions until 1 month after the last case, droplet precautions were implemented, ill residents were isolated, and group activities were canceled. During the outbreak, a total of 55 persons experienced illnesses that met the case definition; 12 were hospitalized, and seven died. PCR detected *Mycoplasma pneumoniae* DNA in 40% of specimens. *M. pneumoniae* should be considered a possible cause of respiratory illness outbreaks in long-term care facilities. Morbidity and mortality from respiratory disease outbreaks at long-term care facilities might be minimized if facilities monitor for respiratory disease clusters, report outbreaks promptly, prioritize diagnostic testing in outbreak situations, and implement timely and strict infection control measures to halt transmission.

## Epidemiologic Investigation

The facility has 152 beds and includes an Alzheimer disease locked unit, a skilled nursing facility, and assisted living wings. A medical director and private physicians provide residents with medical care. At the time of the outbreak, the facility had 143 residents and 132 staff members. On June 20, the facility alerted ECDHD, which then alerted NDHHS, that they had 22 residents ill with a respiratory illness of unknown etiology. The outbreak had started in the Alzheimer unit, where, on June 2, the index patient experienced fever and cough. He was examined in his primary care provider’s office on June 4, and on June 5 was started on amoxicillin. He was not hospitalized and died on June 7.

Three other Alzheimer unit residents became ill, and by the time ECDHD and NDHHS were notified, the illness had spread to other units. No diagnostic specimens had been collected. On June 22, NDHHS established a working case definition and instructed the facility to ask the attending physician to collect NP and OP swabs from a hospitalized resident and asked the facility to collect specimens from any other residents or staff members with fever ≥100.4°F (≥38.0°C) and cough, or a diagnosis of pneumonia. However, specimen collection was delayed 2 days until trained ECDHD staff members visited the facility. ECDHD informed community physicians of the outbreak and requested notification of any unexplained pneumonia cases.

On June 28, NDHHS received the first laboratory report of a specimen found to test positive for *M. pneumoniae*. On July 1, NDHHS distributed a health alert to primary care providers, infectious disease personnel, urgent care centers, and public health departments in eight affected and adjacent counties to facilitate case finding, advise the medical community of the outbreak and suspected etiology, and provide guidance regarding treatment of suspected cases. Fluoroquinolones, tetracyclines, and macrolides were recommended by NDHHS as treatment options, with levofloxacin (a fluoroquinolone) preferred based on early reports of treatment failure with macrolides (Figure). Under a new surveillance case definition, health care providers were asked to notify ECDHD of patients who had a fever ≥100.4°F (≥38.0°C), a diagnosis of pneumonia by clinical examination or chest radiograph, and an epidemiologic link to the facility. ECDHD collected NP and OP specimens from these persons if they agreed to testing. Autopsy specimens were obtained from one decedent. At the time of specimen collection, clinical and demographic information was gathered about each affected patient (Table). Specimens were sent to the Nebraska Public Health Laboratory (NPHL) initially, and then to CDC. At CDC, multiplex, real-time PCR testing was performed for *M. pneumoniae, Chlamydophila pneumoniae, Legionella* species, and human nucleic acid (as a control) (1).

After the likely etiologic agent was identified as *M. pneumoniae*, the case definition was modified. A probable case was defined as an acute respiratory illness and either a fever ≥100.4°F (≥38.0°C) or a pneumonia diagnosed by radiograph in a person with an epidemiologic link (i.e., resident, staff member, staff family member, or visitor). A confirmed case was defined as illness meeting the probable case definition plus a PCR test result positive for *M. pneumoniae* on an NP or OP swab or autopsy specimen during June 2–August 18. Thirty-five of 55 (64%) persons whose illness met the probable case definition were sampled; 14 of the 35 (40%) sampled patients were positive for *M. pneumoniae* by PCR. Because of community concern, 37 patients whose illness did not meet the case definition also were sampled; five (14%) had test results positive for *M. pneumoniae.* However, three of the five did not meet the clinical criteria, and two had no epidemiologic link to the facility; the five were excluded from analysis.

Of the 55 probable and confirmed cases of *M. pneumoniae*, 20 (36%) were in facility residents; 22 (40%) were in staff members; and 13 (24%) were in community members. Ten (50%) of the residents were hospitalized, and seven (35%) died (one of the patients who died was not hospitalized); two (15%) community members with an epidemiologic link to the facility were hospitalized, and one (8%) died. No staff member was hospitalized or died. Among the 55, the overall median age was 46 years (range = 2–96 years); among residents, 89 years (range = 62–96); staff members, 36 years (range = 16–56); and community members, 26 years (range = 2–82). Median age of those who died was 82 years (range = 70–92), compared with 43 years (range = 2–96) for those who survived (chi-square, p = 0.02). Forty-two (76%) patients were female. Twenty-three (42%) patients had a chest radiograph, 16 (70%) of whom had findings consistent with pneumonia (Table).

## Public Health Response

On June 24, ECDHD closed the facility to new admissions and suspended group activities. The facility had confined ill residents to their rooms, isolated affected hallways, posted signs requesting no visitors, and implemented droplet precautions (i.e., use of gowns, gloves, and surgical masks). Family members who insisted on visiting were required to abide by droplet precautions. ECDHD monitored compliance with infection control measures. It was recommended that ill residents be moved to one area with the same staff members assigned to that area every day, avoiding movement of staff members between units (i.e., cohorting). However, the facility was unable to fully implement this measure because of staff coverage concerns.

On July 21, on the basis of reports that ill staff members were working, the facility began screening the staff for fever and symptoms of illness when members arrived at work; those who were ill were discharged from duty until afebrile for ≥24 hours. The facility’s national corporate medical director and infection control nurse worked with ECDHD and NDHHS, and on August 2, the facility’s infection prevention consultant performed a site visit. Facility admissions resumed September 14, one month after the last patient’s illness onset date.

### Discussion

Pneumonia is well-documented as a major cause of morbidity and mortality among persons aged >65 years, particularly those residing in nursing homes (2–6); the patients who died during this outbreak were considerably older than those who survived. Risk factors associated with pneumonia among persons living in nursing homes include older age, difficulty in swallowing because of comorbidities (e.g., Parkinson disease or Alzheimer disease), and being bedridden (4,5). This outbreak was caused by *M. pneumoniae*, an atypical bacterial organism. Although atypical organisms account for ≤40% of community-acquired pneumonias (7), previous studies of nursing home–acquired pneumonias have not reported *M. pneumoniae* as a major cause (5,8,9); fatalities from *M. pneumoniae* are uncommon (10). This outbreak was unusual because of the type of facility and the number of fatalities.

Older patients with pneumonia might have falls, confusion, dizziness, or fatigue, without a fever or other classic pneumonia symptom, or they might have serious comorbidities (e.g., underlying lung disease), making case ascertainment difficult (2,3,5). This outbreak began in the Alzheimer unit, where accurate illness histories could not be obtained and where certain patients were receiving nonaggressive care (e.g., patients might not be tested to determine the cause of an illness or receive interventions beyond those needed for comfort). Additionally, certain patients at the facility were in hospice care for other diseases. These factors are common to outbreaks among older persons (3) and resulted in clinicians deferring diagnostic testing early in the outbreak. Certain patients, including the index patient, were treated with antibiotics ineffective against *M. pneumoniae* infection. After the outbreak’s etiology was confirmed, clinicians frequently prescribed antibiotics on the basis of nursing reports of a fever or cough rather than on patient evaluation or diagnostic test results. As a result, some probable cases might not have been *M. pneumoniae* infection, and certain cases might not have been identified because they did not meet the case definition, particularly those in persons who did not have fever.

**What is already known on this topic?**

*Mycoplasma pneumoniae* is an atypical bacterial organism that can be treated with fluoroquinolones, tetracyclines, or macrolides. *M. pneumoniae* usually is not associated with fatalities, and outbreaks are not commonly reported among geriatric populations. However, older persons are at increased risk for death, and diagnosis of *M. pneumoniae* infection can be delayed because older patients, who might have dementia and other comorbidities, often do not have fever or classic pneumonia symptoms.

**What is added by this report?**

During June–August 2014, 41 probable and 14 laboratory-confirmed cases of *M. pneumoniae* were associated with a single long-term care facility. Seven patients died, and the facility was closed to new admissions for a prolonged period. Delayed recognition of the outbreak and of the etiologic agent prolonged the transmission period and delayed effective interventions.

**What are the implications for public health practice?**

Long-term care facilities should consider *M. pneumoniae* during respiratory illness outbreaks. These facilities need to be alert to outbreaks and plan for prompt diagnostic testing, isolation or cohorting of ill residents, and screening of staff members for illness. Facilities can protect their staff members and residents with education regarding monitoring for outbreaks and infection prevention measures. Delayed recognition of an outbreak and determination of the etiologic agent might prolong the transmission period and delay effective interventions.

Negative PCR results in some probable cases might be attributable to the timing of sampling, the difficulty in obtaining NP samples from certain patients, and the circulation at the time of the outbreak of other respiratory viruses that cause similar symptoms. *M. pneumoniae* was not suspected early in this outbreak, which highlights the need at extended care facilities for prompt recognition and reporting of outbreaks, diagnostic evaluation and testing, and implementation of timely and strict infection control measures to prevent morbidity and mortality.

## Figures and Tables

**FIGURE:**
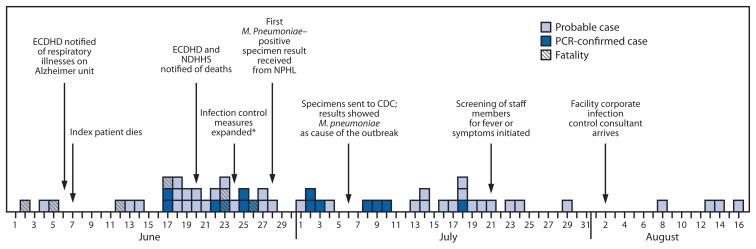
Timeline of *Mycoplasma pneumoniae* outbreak in a long-term care facility — Nebraska, June–August 2014 **Abbreviations:** PCR = polymerase chain reaction; ECDHD = East Central District Health Department; NDHHS = Nebraska Department of Health and Human Services; NPHL = Nebraska Public Health Laboratory. * Specimens collected from all ill residents. Strict droplet precautions implemented, and facility closed to new admissions.

**TABLE:** Number of patients with confirmed or probable *Mycoplasma pneumoniae* respiratory Illness at a long-term care facility, by selected characteristics — Nebraska, 2014

	Confirmed* (n = 14)		Probable† (n = 41)		Total (N = 55)	
			
Characteristic	No.	(%)	No.	(%)	No.	(%)
**Link to facility**						
Resident	6	(43)	14	(34)	**20**	**(36)**
Staff member	5	(36)	17	(41)	**22**	**(40)**
Community member	3	(21)	10	(24)	**13**	**(24)**
**Sex**						
Women	11	(79)	31	(76)	**42**	**(76)**
Men	3	(21)	10	(24)	**13**	**(24)**
**Symptom**						
Fever	10	(71)	39	(95)	**49**	**(89)**
Cough	14	(100)	41	(100)	**55**	**(100)**
Chest congestion	10	(71)	21	(51)	**31**	**(56)**
Sore Throat	8	(57)	15	(37)	**23**	**(42)**
**Chest radiograph**						
No. of patients administered	9	(64)	14	(34)	**23**	**(42)**
No. of findings consistent with pneumonia	8	(89)	8	(57)	**16**	**(70)**
**Outcome** §						
Hospitalized	4	(29)	8	(20)	**12**	**(22)**
Died	2	(14)	5	(12)	**7**	**(13)**
**Antibiotic treatment** ¶						
Levofloxacin	11	(79)	15	(37)	**26**	**(47)**
Azithromycin	1	(7)	7	(17)	**8**	**(15)**
Doxycycline	0	—	3	(7)	**3**	**(5)**
Vancomycin	2	(14)	2	(5)	**4**	**(7)**
Beta-lactams**	2	(14)	13	(32)	**15**	**(27)**
Multiple antibiotics	2	(14)	7	(17)	**9**	**(16)**

* Respiratory illness in a patient with an epidemiologic link to the long-term care facility, a fever >100.4°F (>38.0°C) or a positive chest radiograph finding, and a positive *Mycoplasma pneumoniae* polymerase chain reaction test result from a nasopharyngeal, oropharyngeal, or autopsy specimen.

† Respiratory illness in a patient with an epidemiologic link to the long-term care facility, a fever >100.4°F (>38.0°C) or a positive chest radiograph finding, with no laboratory testing done.

§ One death and two hospitalizations were among visitors; all other deaths and hospitalizations were among residents. There were no hospitalizations or deaths among staff members.

¶ The list of antibiotics is not mutually exclusive.

** Included ceftriaxone, piperacillin/tazobactam, amoxicillin clavulanate, amoxicillin, and ampicillin.
